# California Wines and Other Notices

**Published:** 1866-10

**Authors:** 


### CALIFORNIA WINES.

We notice that the California wines of Perkins, Stern & Co. are rapidly growing in favor. At the State Agricultural Fair held in this city last week, a silver medal was awarded to the firm, for the best specimens of California wine that were exhibited.

The committee of the Chicago Medical Society, consisting of Drs. Reid, Paoli, Marguerat, T. D. Fitch, W. E. Clarke, Hurlbut, and Blake, to whom the subject was referred, united in the following report:

WHEREAS, The profession have for a long time experienced the utmost difficulty, amounting almost to impossibility, in procuring for their patients, *pure wines*, as medicinal agents; any movement tending toward supplying this great want, deserves the encouragement of this society:

Your committee having examined the wines presented, and also the evidences of their purity and general excellence from many eminent members of the profession, submitted by the agent, unanimously report the following resolution:

"The California wines from Perkins, Stern & Co., of New York, presented before this Society, by their purity and general excellence are worthy of our confidence and patronage, and we hereby commend them to the medical profession."

---

DR. EDWIN POWELL has been appointed Adjunct Professor of Surgery in Rush Medical College.

Dr. Powell has long been connected with the Institution as Demonstrator of Anatomy. Last year he delivered an instructive course of lectures on Dislocations and Fractures. He brings to his new position a rich experience in civil as well as military surgery. His enthusiastic devotion to his profession, and other excellent qualities as a teacher, cannot fail to secure for his appointment the general approbation of the profession.

---

Students who desire private medical instruction will be received by Drs. Lackey and Lyman. For particulars, enquire at the office of the *Medical Journal*, 169 Dearborn street.

---

In our next issue we intend to give our readers a complete sketch of the course of cholera in Chicago during the present season. The number of cases has been comparatively small, and is rapidly approaching a minimum.

---

We would refer our readers to the advertisement of Mr. Dewar, the agent for Bass & Co.'s celebrated Ale. Those who wish to recommend ale to their patients, will do well to examine the quality of the article offered for sale by Mr. Dewar.

---

Prof. Brainard arrived in this city, on the 21st September, with health much improved by his recent tour in Europe.

